# Diagnosis of Idiopathic Pulmonary Fibrosis in a Possible Usual Interstitial Pneumonia Pattern: a meta-analysis

**DOI:** 10.1038/s41598-018-34230-z

**Published:** 2018-10-26

**Authors:** Heekyung Kim, Soon Ho Yoon, Hyunsook Hong, Seokyung Hahn, Jin Mo Goo

**Affiliations:** 1 Department of Radiology, Seoul National University College of Medicine, Seoul, Korea; 2 Institute of Radiation Medicine, Seoul National University Medical Research Center, Seoul, Korea; 3 Medical Research Collaborating Center, Seoul National University Hospital, Seoul, Korea; 4 Department of Medicine, Seoul National University College of Medicine, Seoul, Korea

## Abstract

This study aimed to determine whether a surgical lung biopsy is essential for IPF diagnosis with the possible UIP CT pattern. We performed literature searches of the MEDLINE and EMBASE databases and included studies that conducted a radiologic-pathologic evaluation of IPF according to the 2011 guideline. Outcomes were pooled using a random-effects model. Twelve studies were included. Pooled proportions of IPF for a UIP pattern were 99% (95%CI, 93% to 100%; I^2^ = 51.7%) and for a possible UIP pattern were 94% (scenario inclusive of probable IPF; 95%CI, 87% to 99%; I^2^ = 82.9%) and 88% (scenario exclusive of probable IPF; 95%CI, 79% to 95%; I^2^ = 82.7%). The pooled percentage difference in the proportion of IPF between the UIP and possible UIP patterns was −2% (95%CI, −4% to 1%; I^2^ = 0.0%) in the former scenario and 4% (95%CI, 0% to 8%; I^2^ = 0.1%) in the latter scenario. The proportion of IPF with the possible UIP pattern was moderately correlated with the prevalence of IPF (correlation coefficient, 0.605; 95%CI, 0.550–0.860). There was a negligible pooled percentage difference in the proportion of IPF between the UIP and possible UIP patterns, indicating that IPF diagnosis can be confirmed without biopsy in suspected IPF cases with the possible UIP pattern.

## Introduction

Idiopathic pulmonary fibrosis (IPF) is a specific form of chronic, progressive fibrosing interstitial pneumonia of unknown cause characterized by worsening dyspnea and lung function^1^. With the recent introduction of anti-fibrotic agents for IPF treatment including nintedanib and pirfenidone, accurate diagnosis of IPF has become more important^2,3^. Traditionally, IPF has been confirmed with a histopathologic evaluation of a lung biopsy specimen but the role of high-resolution computed tomography (HRCT) has recently become more central in the diagnostic pathway of IPF^4,5^. The official ATS/ERS/JRS/ALAT statement in 2011 specified the HRCT patterns of usual interstitial pneumonia (UIP) into 3 diagnostic categories^1^: UIP pattern, if honeycombing and reticular abnormality with subpleural, basal predominance exists without features inconsistent with the UIP pattern; possible UIP pattern, if honeycombing is absent but the imaging features otherwise meet the criteria for the UIP pattern; and inconsistent with UIP pattern. IPF can be diagnosed without a pathologic confirmation in patients with the UIP pattern on HRCT scan because it exclusively indicates IPF, while a surgical lung biopsy is recommended for the possible or inconsistent with UIP patterns.

Recent studies have shown that basilar predominant reticular opacities without honeycombing on HRCT were strongly associated with pathologic UIP^6,7^. Surgical lung biopsy inevitably accompanies morbidity and mortality, especially for nonelective procedures (in-hospital mortality, 16%)^8^. If a reticular abnormality with subpleural basal predominance is highly indicative of IPF regardless of presence of honeycombing, a surgical lung biopsy can be omitted in patients with the possible UIP pattern^9^ as with the UIP pattern. Indeed, the Fleischner society recently proposed the introduction of probable UIP CT pattern which substitutes for possible UIP CT pattern in the 2011 guideline and the allowance of IPF diagnosis without surgical lung biopsy in case of probable UIP CT pattern based on a selective review of few number of relevant studies. Thus, we performed a meta-analysis of studies to determine whether the surgical lung biopsy is essential for IPF diagnosis in patients with the possible UIP pattern on HRCT scan.

## Results

### Literature search

**Figure 1:**
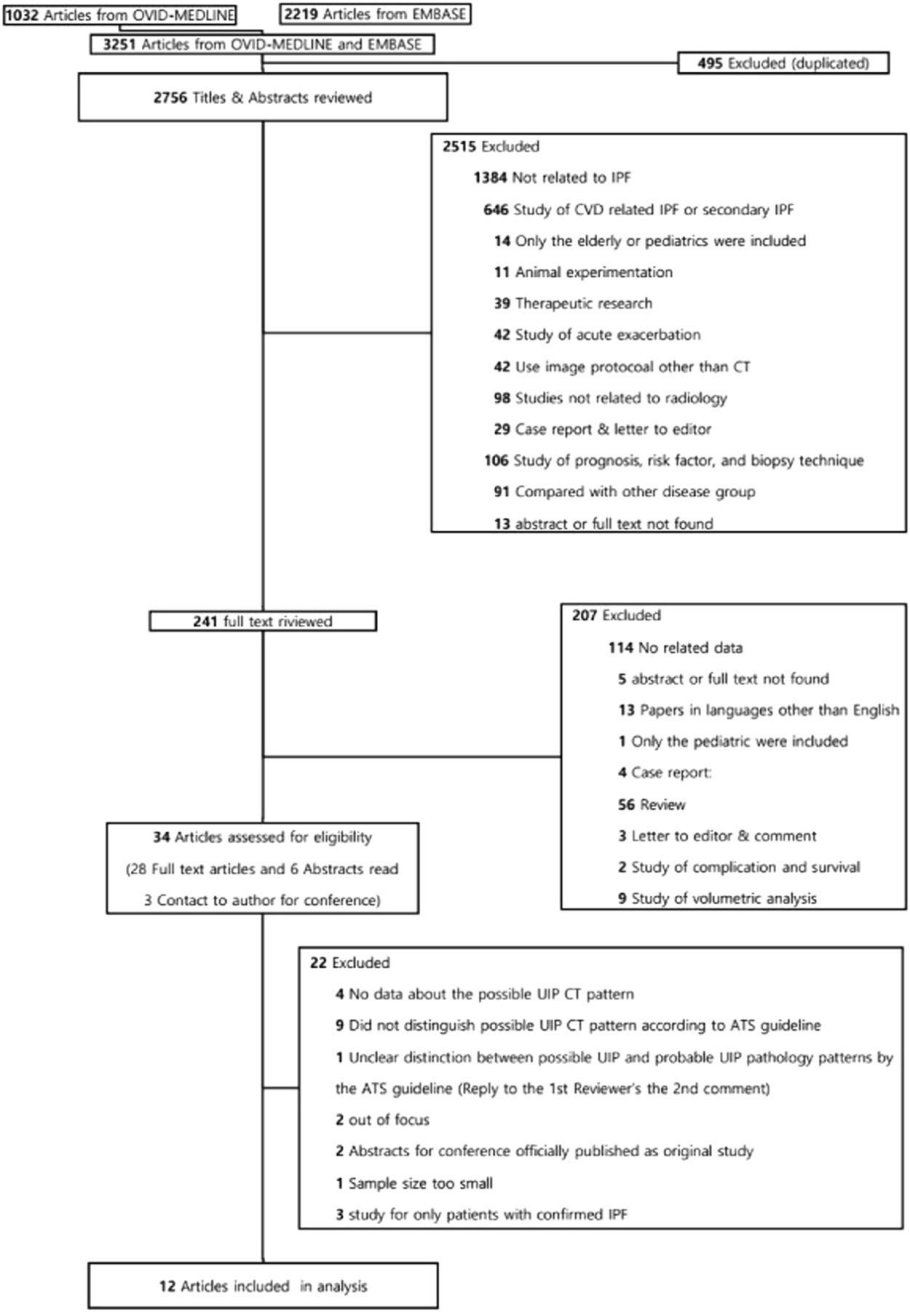
Flow diagram of the literature search.

Of 3251 references identified during our initial database search, 12 studies^7,10–20^ with 13 study populations were finally included in our analysis (Fig. 1 and Table 1). Two study populations for the training and validation datasets were separately extracted in the study of Brownell *et al*.^20^.

**Table 1:** Baseline characteristics of included studies, IPF prevalence, and IPF proportion according to HRCT patterns.

Source		Characteristics of included patients							IPF prevalence		Proportion of IPF in HRCT patterns‡		
		Recruitment year	Country	Patient number	Study design	Age (years)	Sex (M:F)	Biopsy rate	Inclusion of all patients with UIP HRCT pattern	Estimated prevalence	UIP	Possible UIP with inclusion of probable IPF [Exclusion of probable IPF]	Inconsistent with UIP
Ogawa *et al.*^10^		1995 to 2010	Japan	52	Retrospective	Median, 72; range, 32–85	41:11	All	Yes	61.5% (35/52)	15/16 (93.75%)	20/21 (95.2%) [17/21 (81.0%)]	0/15 (0%)
Tomassetti *et al.*^11^		2001 to 2008	Italy	64	Prospective	Unknown		All	No	62.5% (40/64)		37/44 (84.1%) [37/44 (84.1%)]	3/20 (15%)
Casoni *et al.*^12^		2011 to 2012	Italy	69	Prospective	Unknown		All	No	63.7% (44/69)		21/22 (95.4%) [18/22 (81.8%)]	23/47 (48.9%)
Raghu *et al.*^7^		2009 to 2010	America, Europe, and Australia	315	Retrospective	Range, 40–80	Unknown	All	No	85.1% (268/315)	109/111 (98.2%)	83/84 (98.8%) [79/84 (94.0%)]	76/120 (63.3%)
Sumikawa *et al.*^13^		1989 to 2006	Japan	114	Retrospective	Mean, 61; range, 25–86	79:35	All	Yes	65.7% (75/114)	16/17 (94.1%)	23/24 (95.8%) [23/24 (95.8%)]	36/73 (49.3%)
Chung *et al.*^14^		1999 to 2010	USA	201	Retrospective	Mean, 62.9± 10.2	125:76	All	Yes	62.1% (125/201)	20/25 (80.0%)	84/106 (79.2%) [67/106 (63.2%)]	21/70 (30%)
Hanley *et al.*^15^		2012 to 2014	UK	104	Retrospective	Unknown	Unknown	15/53 †	Yes	60.5% (63/104)^‡‡^		12/15 (80%) [12/15 (80%)]	
Kaunisto *et al.*^16^		2012	Finland	123	Retrospective	Mean, 73.5	74:49	24/123^††^	Yes	79.6% (98/123)^‡‡^	9/10 (90%)	7/7 (100%) [7/7 (100%)]	4/7 (57.1%)
Pezzuto *et al.*^17^		Unknown	Italy	124	Prospective	Mean, 69.0±7.9	87:37	23/124^†††^	Yes	79.8% (99/124)^‡‡^		7/7 (100%) [7/7 (100%)]	16/16 (100%)
Bondue *et al.*^18^		2015 to 2016	Belgium	30	Prospective	Median, 62; range, 26-80	14:16	All	No	40.0% (12/30)		5/5 (100%) [5/5 (100%)]	6/24 (25%)
Yagihashi *et al.*^19^		2007 to 2012	USA	241	Retrospective	Mean, 65.7± 8.0	184:57	All	Yes	90.1% (219/241)	100/102 (98.0%)	64/64 (100%) [60/64 (100%)]	55/75 (73.3%)
Brownell *et al.*^20^	Derivation cohort	2002 to 2015	USA	385	Retrospective	Mean, 60	174:211	All	No	38.2% (166/434)^‡‡‡^		44/64 (68.5%) [40/64 (62.5%)]	73/321 (22.7%)
	Validation cohort	1999 to 2016	USA	166	Retrospective	Mean, 64	97:70	All	No	95.2% (181/190)^‡‡‡^		69/71 (97.1%) [67/71 (94.4%)]	88/95 (92.6%)

*Definition of abbreviation:* HRCT = high resolution computed tomography; IPF = idiopathic pulmonary fibrosis; UIP = usual interstitial pneumonia.

*The prevalence of IPF was presumed to be underestimated because patients with a UIP pattern on HRCT scan were excluded.

**According to the 2011 ATS/ERS/JRS/ALAT guideline, IPF was diagnosed by specific combinations of the HRCT and surgical lung biopsy pattern: UIP pattern on HRCT with any patterns except for not UIP pattern on a surgical lung biopsy; possible UIP pattern on HRCT with a UIP or probable UIP pattern in a surgical lung biopsy. Multidisciplinary discussion is recommended to make a diagnosis of IPF for the following cases that probably or possibly had IPF: probable IPF, possible UIP pattern on HRCT with a possible UIP pattern or unclassifiable fibrosis on surgical lung biopsy; possible IPF, inconsistent UIP pattern on HRCT with a UIP pattern on surgical lung biopsy. Depending on whether the probable IPF was included in the IPF diagnosis or not, our analyses were performed based on the 2 following scenarios: scenario inclusive of probable IPF and scenario exclusive of probable IPF.

^†^Of the 53 patients with possible UIP/NSIP, 15 patients had a lung biopsy.

^††^A surgical lung biopsy was performed in 27 (22%) patients. Among them, three samples were not available for a re-evaluation.

^†††^A surgical lung biopsy was performed in 16 patients with possible UIP pattern or inconsistent with UIP pattern on HRCT scan. The biopsy was additionally performed in 7 patients with reticular opacities and honeycombing on HRCT scan, which evenly distributed from lung apex to basal lung.

^‡^The IPF proportion was calculated by taking the total number of patients undergoing biopsy as the denominator.

^‡‡^The prevalence of IPF was calculated by adding patient data for a definite UIP pattern without lung biopsy.

^‡‡‡^The prevalence of IPF was calculated to include data from definite UIP pattern without lung biopsy without inclusion in the study.

### Characteristics and quality assessment of the included studies

The number of the study populations in the included studies ranged from 30 to 385. Studies were performed in Europe, America, and Asia. The median or mean age of the patients was 64 years. The median portion of male patients was 62.1%. Surgical lung biopsy was performed on all patients in 9 studies, and in the 3 remaining studies, the biopsy was performed in a portion of patients. Seven studies^7,10,13,14,16,18,19^ included patients with a UIP pattern on HRCT scan, while 5 studies^11,12,15,17,20^ excluded those patients. In the former studies, the prevalence of IPF ranged from 40% to 91% with a median of 67.3%, and in the latter studies, the prevalence of IPF ranged from 38% to 95% with a median of 63.1% (Table 1).

When assessed by the QUADAS-2 tool, the included studies appeared to have relatively low risks of bias in patient selection but unclear in index test, reference standard, and in flow and timing (Supplementary Data 1). In the domain of patient selection, the study population was consecutively included in most of the included studies. However, in the domain of index test, it was unclear whether the index test was evaluated without knowing the result of the reference standard in several studies. It was also unclear whether the reference standard results were interpreted without a-priori knowledge of the results of the index test. In flow and timing, the interval between the index test and the reference standard was not clearly described. Overall, the methodological quality of the literature was low because the risk of bias in the QUADAS-2 tool was found to be “Unclear” in more than half of the studies. Concerns about applicability in individual studies were assessed as relatively low.

### IPF proportion according to HRCT pattern

**Figure 2:**
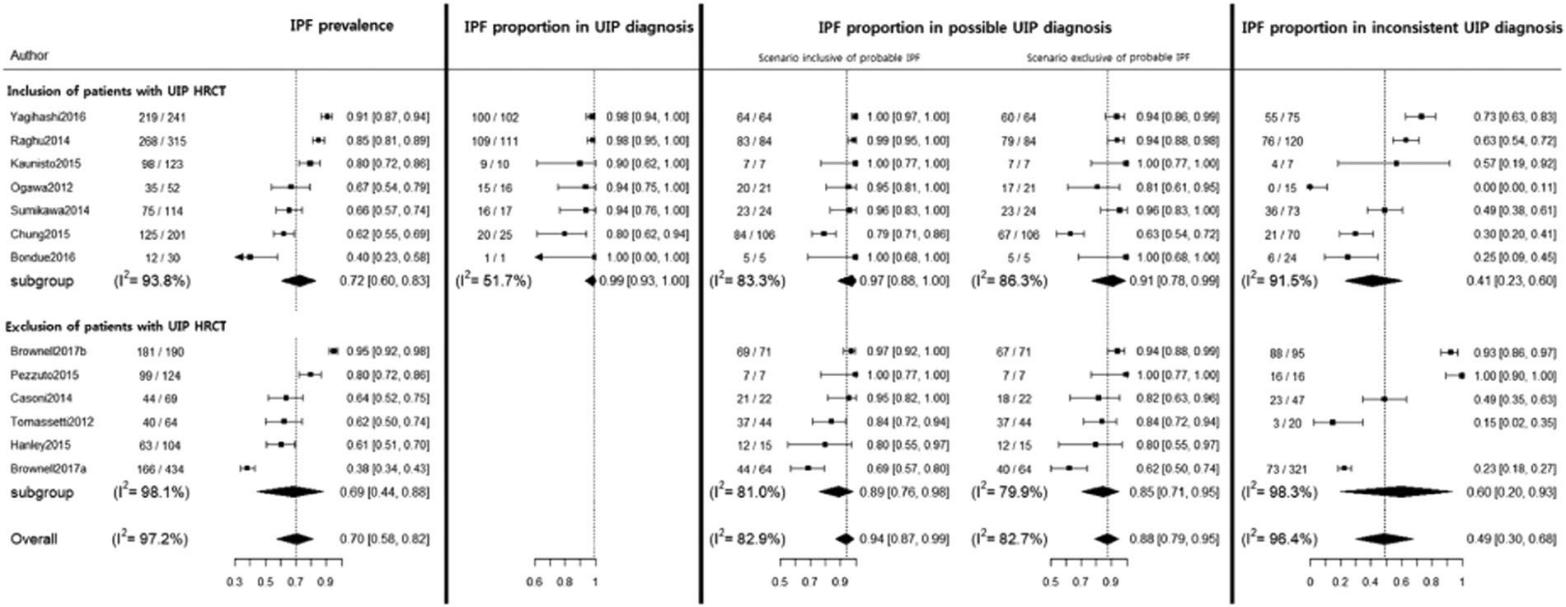
Proportion of idiopathic pulmonary fibrosis when biopsied according to high resolution computed tomography patterns of usual interstitial pneumonia. *The probable IPF was defined as the combination of possible UIP radiology pattern on HRCT scan and possible UIP pathology pattern or unclassifiable fibrosis on surgical lung biopsy according to the official ATS/ERS/JRS/ALAT statement in 2011.

The pooled proportion of IPF for the UIP pattern was 99% (95%CI, 93% to 100%; I^2^ = 51.7%). Pooled proportions of IPF for the possible UIP pattern were 94% (95%CI, 87% to 99%; I^2^ = 82.9%) in the scenario inclusive of probable IPF, and 88% (95%CI, 79% to 95%; I^2^ = 82.7%) in the scenario exclusive of probable IPF. When compared between studies with a UIP pattern included and those with it excluded (Fig. 2), the proportion of IPF tended to be higher in the former studies than in the latter studies regardless of scenario. The proportion of IPF for an inconsistent UIP pattern was too heterogeneous to be meta-analyzed, with a range from 0% to 100% (median, 49%).

### Percent difference in IPF proportion between UIP pattern and possible UIP patterns

**Figure 3:**
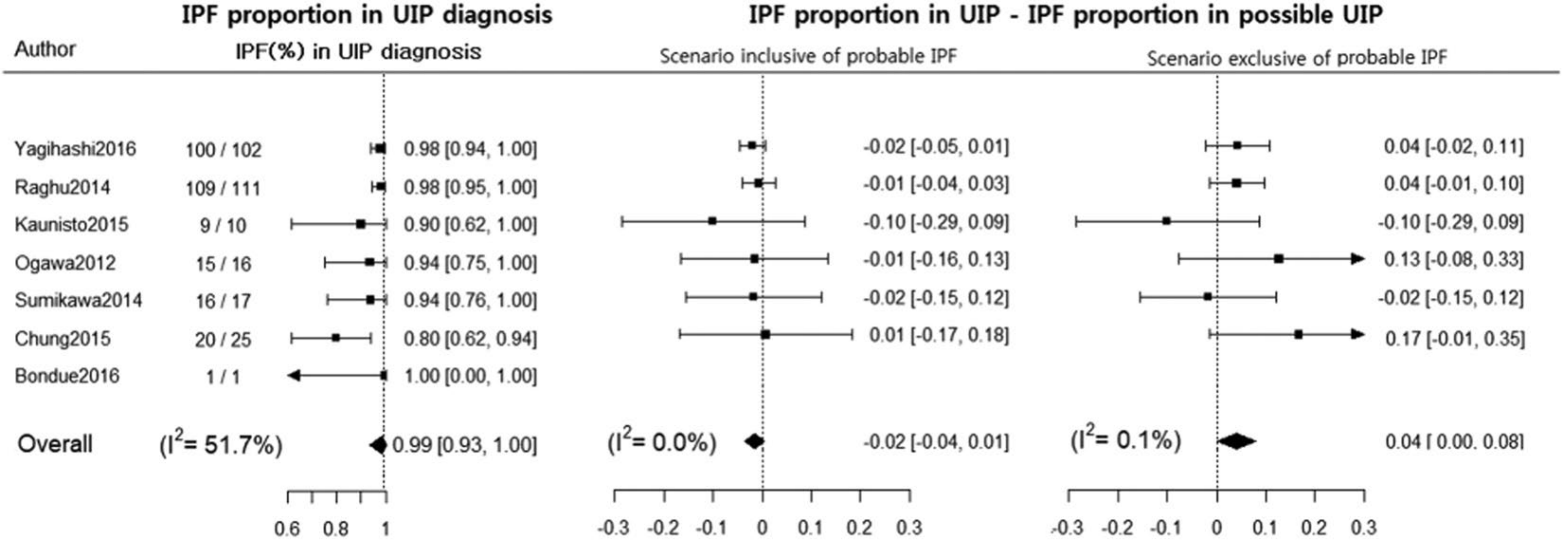
Percentage difference in the proportion of idiopathic pulmonary fibrosis between usual interstitial pneumonia and possible usual interstitial pneumonia patterns on high resolution computed tomography scan without zero-cell corrections. *The probable IPF was defined as the combination of possible UIP radiology pattern on HRCT scan and possible UIP pathology pattern or unclassifiable fibrosis on surgical lung biopsy according to the official ATS/ERS/JRS/ALAT statement in 2011.

The pooled difference in proportions of IPF between the UIP and possible UIP patterns was −2% (95%CI, −4% to 1%; I^2^ = 0.0%) in a scenario inclusive of probable IPF and 4% (95%CI, 0% to 8%; I^2^ = 0.1%) in a scenario exclusive of probable IPF without zero-cell corrections (Fig. 3). This finding did not include Bondue’s^18^ study including zero cells in both the UIP pattern and the possible UIP pattern.

When the correction was performed by adding 0.5 to the frequency of studies containing 0 cells, the difference in proportions of IPF between the UIP and possible UIP pattern was −1% (95%CI, −4% to 1%; I^2^ = 0.0%) in the scenario inclusive of probable IPF and 4% (95%CI, 0% to 8%; I^2^ = 0.0%) in the scenario exclusive of probable IPF (Supplementary Data 2).

### Relationship between IPF prevalence and IPF proportion when biopsied

**Figure 4:**
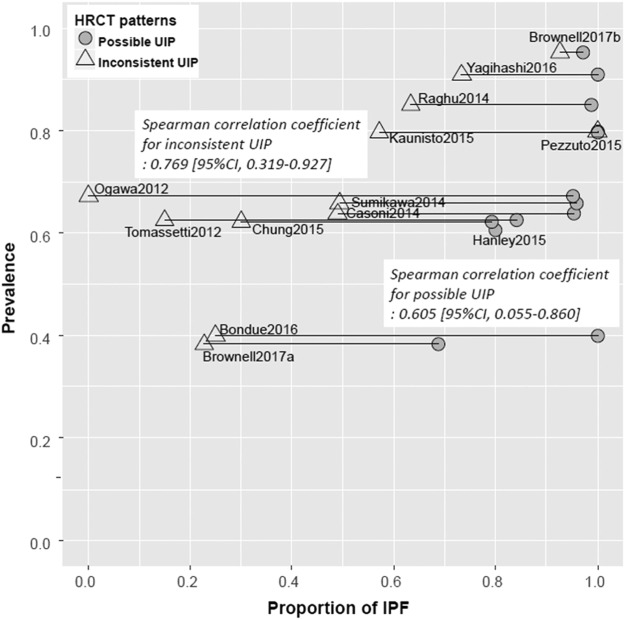
Relationships between the prevalence and proportion of idiopathic pulmonary fibrosis when biopsied in possible usual interstitial pneumonia and inconsistent usual interstitial pneumonia patterns.

In case with a UIP pattern, IPF prevalence was not correlated with the proportion of IPF (Spearman correlation coefficient, 0.07; 95%CI, −0.72 to −0.78). However, in cases with possible and inconsistent UIP patterns, IPF prevalence and the proportion of IPF showed a moderate monotonically increasing relationship (Spearman correlation coefficient, 0.605; 95%CI, 0.055 to 0.860 for a possible UIP pattern; 0.769; 95%CI, 0.319 to 0.928 for an inconsistent UIP pattern) (Fig. 4 and Supplementary Data 3).

### Assessment of publication bias

Funnel plot asymmetry was assessed for proportions of IPF in a possible UIP pattern and an inconsistent UIP pattern, which were presented in 13 and 12 study populations, respectively. No obvious asymmetries were observed and P values for Egger’s test were 0.6131 and 0.7268 (Supplementary Data 4).

## Discussion

This meta-analysis revealed that a pooled percentage difference in proportions of IPF diagnosis was negligible between the CT UIP and possible UIP pattern: −2% (95%CI, −4% to 1%) if possible UIP cases probably having IPF actually had IPF in 100% of cases (scenario inclusive of probable IPF). When we assumed that possible UIP cases probably having IPF were not IPF at all, the pooled percentage difference between the patterns was marginal (4%; 95%CI, 0% to 8%) (scenario exclusive of probable IPF). The pooled proportion of IPF in the possible UIP pattern based on the former and latter assumptions were 94.0% (95%CI, 87% to 99%) and 88.0% (95%CI, 79% to 95%), respectively, and this depended on IPF prevalence (correlation coefficient, 0.605; 95%CI, 0.055–0.860).

These results imply that IPF can be confidently diagnosed solely based on the HRCT finding in patients with a possible UIP pattern without surgical lung biopsy, if IPF suspects were appropriately identified. This is totally in accord with the latest recommendation of the Fleischner Society on the diagnostic criteria of IPF^21^. The society introduced a probable UIP pattern as a new category by upgrading the possible UIP pattern and proposed the IPF diagnosis without surgical lung biopsy in patients who had a typical clinical context of IPF with UIP or probable UIP CT pattern. Our systematic review of all relevant publications validated their recommendation.

Our finding suggests that radiologic honeycombing is not mandatory for the HRCT diagnosis of IPF. The radiologic honeycombing is one of a spectrum of CT findings, representing the late stage of IPF. It represents advanced fibrotic lung changes which contain clustered cystic airspaces with thick fibrous walls, along with a complete loss of acinar architecture^1^. Prior to overt honeycombing, radiologic reticular abnormalities originate from fibrotic thickening of a distorted interstitium and formation of small cysts, which are often beyond the resolution of HRCT and have presumably progressed into honeycombing^22^. Accordingly, reticular opacities, which typically have a basilar, subpleural predominance on HRCT can correspond to an earlier CT finding of overt radiologic honeycombing^23^. Indeed, nintedanib had similar therapeutic effects in patients with suspected IPF not only with a UIP pattern but also those with a possible UIP pattern on HRCT scan^24^.

IPF prevalence was moderately correlated with the pooled proportion of IPF in the cases with a possible UIP pattern. This suggests that assessment of the degree of suspicion of IPF is crucial in patients with a possible UIP pattern before IPF diagnosis^20^. The degree of suspicion may be assessed qualitatively by clinicians or quantitatively by the pretest probability model which consists of age, gender, and traction bronchiectasis score on HRCT scan^20^. Both assessments require sufficient experience with IPF diagnosis, which is why multidisciplinary discussion between experts is emphasized in the current guideline^1^. In the setting where IPF is highly suspected by experienced clinicians or the pretest probability model, we believe that IPF is sufficiently diagnosed with a possible UIP CT pattern without biopsy^21^.

The pooled proportion of IPF was 99.0% (95%CI, 93% to 100%) in patients with a UIP CT pattern, supporting the current guideline, which recommends making a diagnosis of IPF without biopsy in those patients. On the other hand, for an inconsistent UIP HRCT pattern, the proportion of IPF varied from 0% to 100% across studies, and it was strongly affected by IPF prevalence (correlation coefficient, 0.769; 95%CI, 0.319–0.928). It is appropriate that the current guideline recommends surgical lung biopsy for patients having the inconsistent UIP HRCT pattern.

Our study had several limitations. First, as multidisciplinary discussion was not performed in this study, so two different assumptions were introduced for handling probable IPF cases (possible UIP pattern or unclassifiable fibrosis on a surgical lung biopsy): all the cases were IPF (scenario inclusive of probable IPF) or none were IPF (scenario exclusive of probable IPF). In the clinical setting where multidisciplinary discussion is introduced, we may expect a percentage difference in proportions of IPF diagnosis of −2% (scenario inclusive of probable IPF) to 4% (scenario exclusive of probable IPF) between the radiologic UIP and possible UIP patterns. Second, the study population was defined differently across studies, especially with regard to the inclusion of patients with a radiologic UIP pattern. The prevalence in some of the included studies was underestimated due to the exclusion of patients with typical UIP CT pattern from the study population, and this might have affected the result of correlation analysis between IPF prevalence and the proportion of IPF when biopsied. Likewise, the proportion of IPF in a certain UIP CT pattern might be incorrectly assessed in 4 studies where the confirmatory surgical biopsy was performed in a small portion of IPF patients. The percentage difference of IPF diagnosis between the UIP and possible patterns could be pooled in 6 of 13 included study population in Table 1 due to lack of information about IPF diagnosis in the UIP pattern. Thus, our result requires a further validation in a large prospective setting. Third, the potential inter-observer variability on the categorization of HRCT patterns could not be considered in our analyses. Fourth, the search list was limited and only two databases were used for searching, so it is possible that some relevant studies might have been missed. Fifth, among the included studies, UIP patterns on HRCT scan were mainly analyzed by academic radiologists. Accordingly, our result needs to be cautiously interpreted in the setting of a community center and there may be significant disagreements in applying our result to physician-based community, as suggested by Flaherty *et al*.^25^ Sixth, there was substantial heterogeneity in the IPF proportion with possible and inconsistent UIP diagnoses, presumably because the IPF prevalence in each study was different. In order to resolve this heterogeneity, we pooled the percentage difference of IPF diagnosis between the UIP and possible patterns and the corresponding I-square was 0.0% to 0.1% in Fig. 3. Seventh, even though the brief summary protocol was shared across the authors before the beginning of study, we didn’t have a published protocol.

In conclusion, our meta-analysis suggests that the pooled percentage difference in the proportions of IPF diagnosis was negligible between radiologic UIP and possible UIP patterns, indicating that IPF diagnosis can be made with a possible UIP pattern without biopsy just as with a UIP pattern in patients highly suspected of having IPF. This enables maintaining an accurate diagnosis while avoiding the morbidity and mortality of surgical lung biopsy in IPF patients having a possible UIP CT pattern who could potentially benefit from anti-fibrotic agents.

## Methods

### Search strategy

Two of the authors (K.H.K. and S.H.Y.) independently performed a literature search of the OVID/MEDLINE and EMBASE database to identify relevant publications that conducted a radiologic-pathologic evaluation according to the ATS/ERS/JRS/ALAT guideline in 2011. We used keywords related to ‘idiopathic pulmonary fibrosis’, ‘computed tomography’, and ‘pattern’ from January 2010 until May 2017 (Supplementary Data 5). Searches were limited to English-language publications and human studies. This search was further supplemented by screening bibliographies of the retrieved articles and review articles.

### Selection of studies

We applied the following criteria to determine eligibility: (i) study populations consisting of 5 or more patients who were suspected of having interstitial lung disease and underwent a surgical lung biopsy; (ii) a radiologic-pathologic evaluation of IPF based on the combination of HRCT and surgical lung biopsy patterns in accordance with the 2011 statement; (iii) data presented in sufficient detail to assess the proportion of IPF in the possible UIP pattern on HRCT scan.

We excluded studies where the proportion of IPF in each HRCT pattern was not extractable due to exclusive inclusion of IPF without inclusion of any other interstitial lung diseases, and studies dealing with patients with specified causes of interstitial lung disease (e.g., domestic and occupational environmental exposures, connective tissue disease, and drug toxicity). If study populations were overlapping between studies, the study having enrolled the largest number of patients was included. Review articles, editorials, letters, case reports, and guidelines for management were excluded. When necessary, we contacted the author for additional data related to the full-text article.

### Assessment of outcomes

The primary outcome of our meta-analysis was a percentage difference in the proportion of IPF diagnosis between the UIP and possible UIP patterns on HRCT scan. Secondary outcomes included the proportion of IPF diagnosis and the correlation between prevalence of IPF and proportions of IPF diagnosis according to the 3 HRCT patterns.

According to the 2011 ATS/ERS/JRS/ALAT guideline, HRCT could have any of the 3 aforementioned patterns and the result of histopathologic examination of surgical lung biopsy encompassed the following 5 patterns: UIP pattern, probable UIP pattern, possible UIP pattern, unclassifiable fibrosis, and non-UIP pattern. IPF was diagnosed with specific combinations of the HRCT and histopathologic patterns (Supplementary Data 6): UIP pattern on HRCT with any pattern except for the non-UIP pattern on surgical lung biopsy or possible UIP pattern on HRCT with a UIP or probable UIP on a surgical lung biopsy.

Multidisciplinary discussion is recommended to make a diagnosis of IPF for the following cases that probably or possibly had IPF: probable IPF, possible UIP pattern on HRCT with a possible UIP pattern or unclassifiable fibrosis on surgical lung biopsy; possible IPF, inconsistent UIP pattern on HRCT with a UIP pattern on surgical lung biopsy. As the multidisciplinary discussion was not introduced in most of the included studies, we initially assumed that those combinations had IPF in 100% of cases (scenario inclusive of probable IPF). Subsequent analysis was also performed to explore the results as if none of those cases had IPF (scenario exclusive of probable IPF).

The prevalence of IPF potentially affects the proportion of IPF when biopsied, so we calculated the prevalence of IPF in the included studies. As patients with a radiologic UIP pattern are not subjected to surgical lung biopsy in the current guideline, some studies included those patients, while other studies excluded them from their study populations. The prevalence of IPF was inevitably underestimated in the latter studies because IPF patients with a radiologic UIP pattern were excluded. We also calculated the number of IPF proportion in possible CT UIP pattern using given data in following 3 studies; Ogawa K *et al*.^10^, Casoni *et al*.^12^, and Hanley *et al*.^15^. Two studies were provided with abstracts, so there was insufficient information to obtain IPF proportions, and the other study did not distinguish pathologic probable UIP from possible UIP.

### Data extraction and quality assessment

Data extraction and quality assessment were performed independently by 2 authors (S.H.Y. and K.H.K). Quality assessment of the included literature was conducted based on the Quality Assessment of Diagnostic Accuracy Studies (QUADAS)-2 tool^26^. All disagreements were harmonized by consensus.

### Statistical analysis

The proportion of IPF was pooled via a DerSimonian-Laird random-effect model according to the HRCT patterns. The proportions were double arcsine transformed to stabilize variances and then back-transformed^27^. A pooled difference in the proportion of IPF between the UIP pattern and possible UIP pattern was estimated without zero-cell corrections and excluding a study with zero-cell counts in both patterns. A sensitivity analysis was conducted after zero-cell corrections of adding 0.5 to all cells of the study results tables where zero-cells were observed in one or both patterns in a study. Heterogeneity across the included studies was evaluated using *I*^2^ statistics. *I*^2^ was derived from the Cochran Q statistic with the following equation, *I*^2^ = 100% x (Q-df)/Q. An *I*^2^ statistic >50% was regarded as indicating substantial statistical heterogeneity^28^. Spearman correlation analysis was used to assess a monotonic relationship between IPF prevalence in study group and IPF proportion in each HRCT pattern. Publication bias was assessed by visually inspecting the distribution of observed studies on a funnel plot and Egger’s tests for asymmetry was done to quantify the degree of bias. All meta-analyses were done in R version 3.4.0 using the package *metafor*^29^.

### Informed consent

Written informed consent was not required for this study.

### Ethical approval

Institutional Review Board approval was not required because this meta-analysis does not involve human subjects.

## Electronic supplementary material

Dataset 1, Dataset 2, Dataset 3, Dataset 4, Dataset 5, Dataset 6

## Data Availability

The datasets generated during and/or analysed during the current study are available from the corresponding author on reasonable request.
