# Supplementary material for: Diagnosis of Idiopathic Pulmonary Fibrosis in a Possible Usual Interstitial Pneumonia Pattern: a meta-analysis
Source: Sci Rep. 2018 Oct 26;8:15886. doi: 10.1038/s41598-018-34230-z (PMC6203840; doi:10.1038/s41598-018-34230-z)
Supplement: Supplementary file 1 — Dataset 1, Dataset 2, Dataset 3, Dataset 4, Dataset 5, Dataset 6 [file 41598_2018_34230_MOESM1_ESM.pdf]

Diagnosis of Idiopathic Pulmonary Fibrosis in a Possible Usual Interstitial Pneumonia Pattern: a meta-analysis

Heekyung Kim[1], Soon Ho Yoon[1,2], Hyunsook Hong[3], Seokyung Hahn[4], Jin Mo Goo[1,2]

[1]Department of Radiology, Seoul National University College of Medicine, Seoul, Korea; [2]Institute of Radiation Medicine, Seoul National University Medical Research Center, Seoul, Korea; [3]Medical Research Collaborating Center, Seoul National University Hospital, Seoul, Korea; [4]Department of Medicine, Seoul National University College of Medicine

Correspondence to: Soon Ho Yoon,

Department of Radiology, Seoul National University College of Medicine, Seoul, Korea; Institute of Radiation Medicine, Seoul National University Medical Research Center, Seoul 101, Daehak-ro, Jongno-gu, Seoul, Republic of Korea, 03080, Tel: 82-2-2072-2584, Fax: 82-2-743-6385, E-mail: yshoka@gmail.com

Supplementary information

Supplementary data 1. Quality assessment of included studies

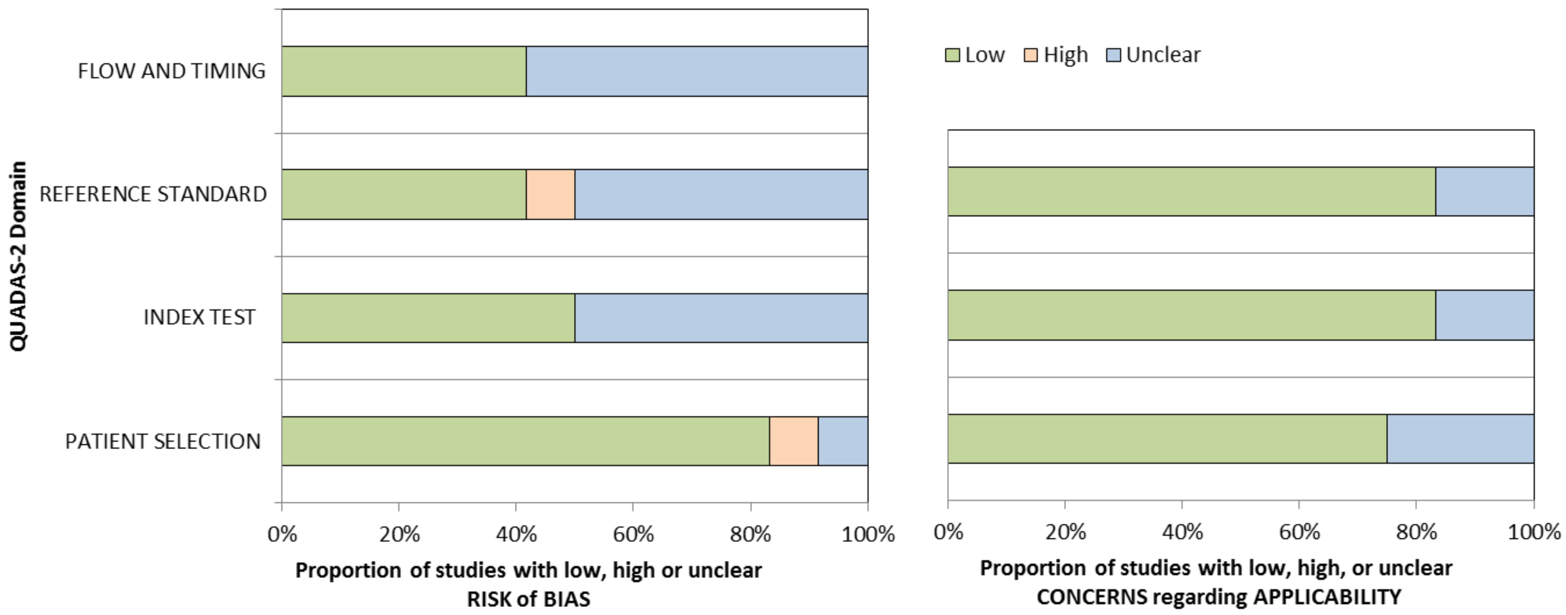

Supplementary data 2. Difference in IPF proportion between UIP pattern and possible UIP pattern with correction by adding 0.5 to the frequency of studies containing 0 cells

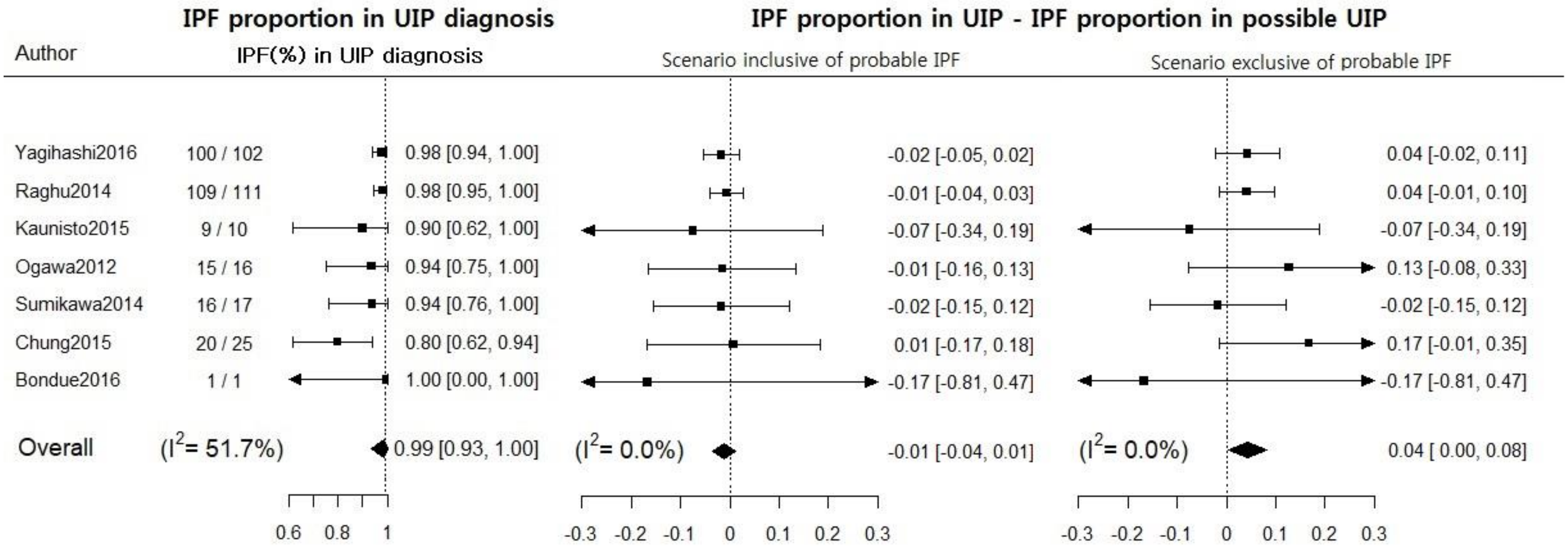

Supplementary data 3. Difference between IPF proportion between possible UIP pattern and inconsistent UIP pattern according to prevalence rate

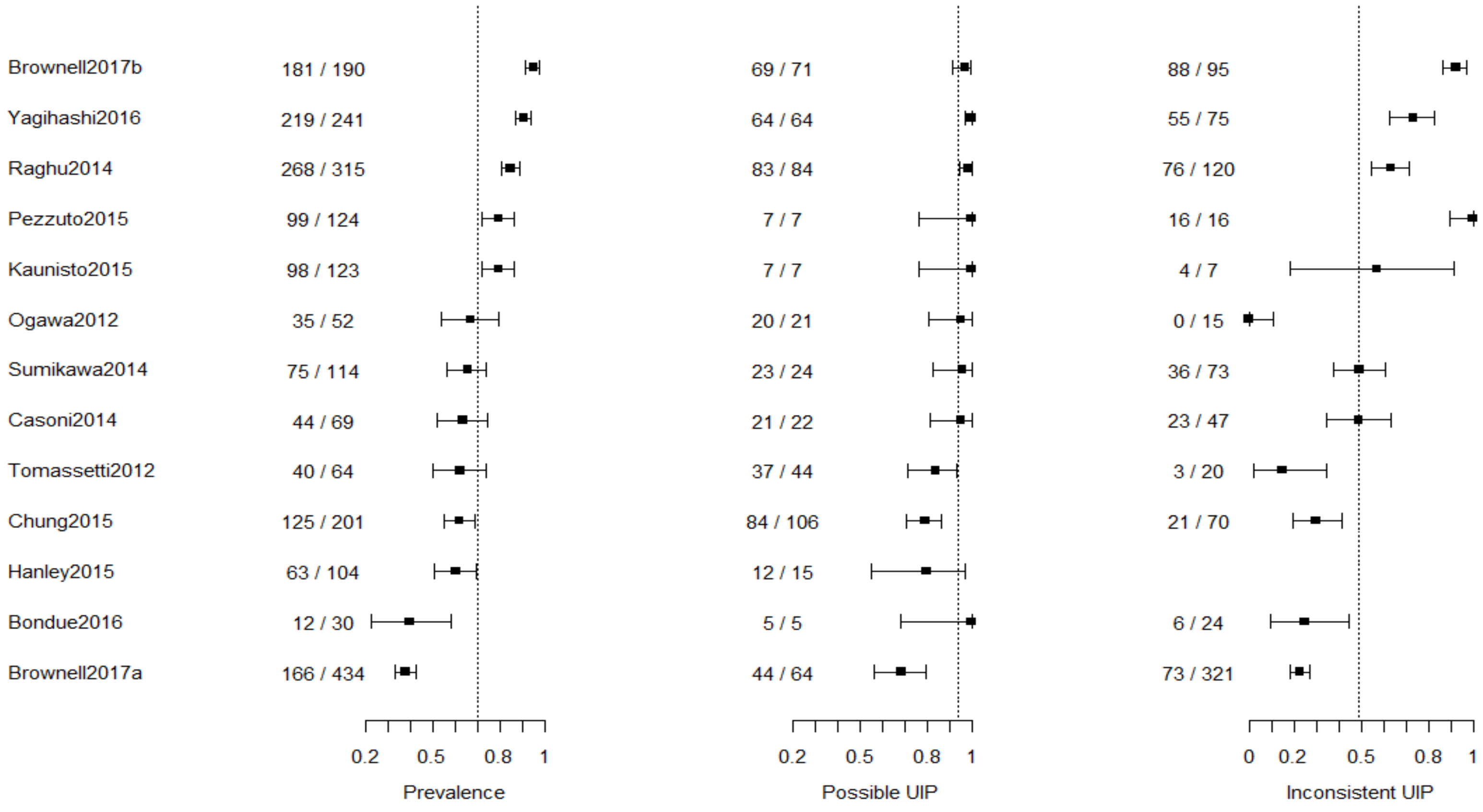

Supplementary data 4. Funnel plot of IPF proportions in possible UIP and inconsistent UIP patterns when biopsied

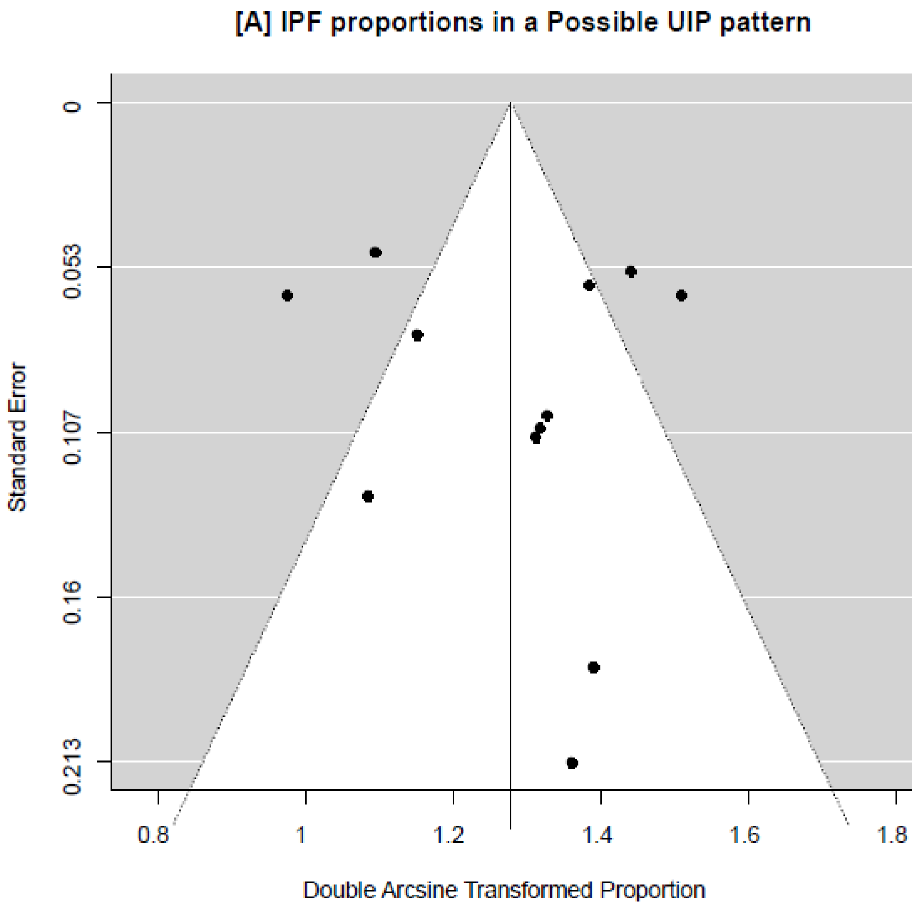

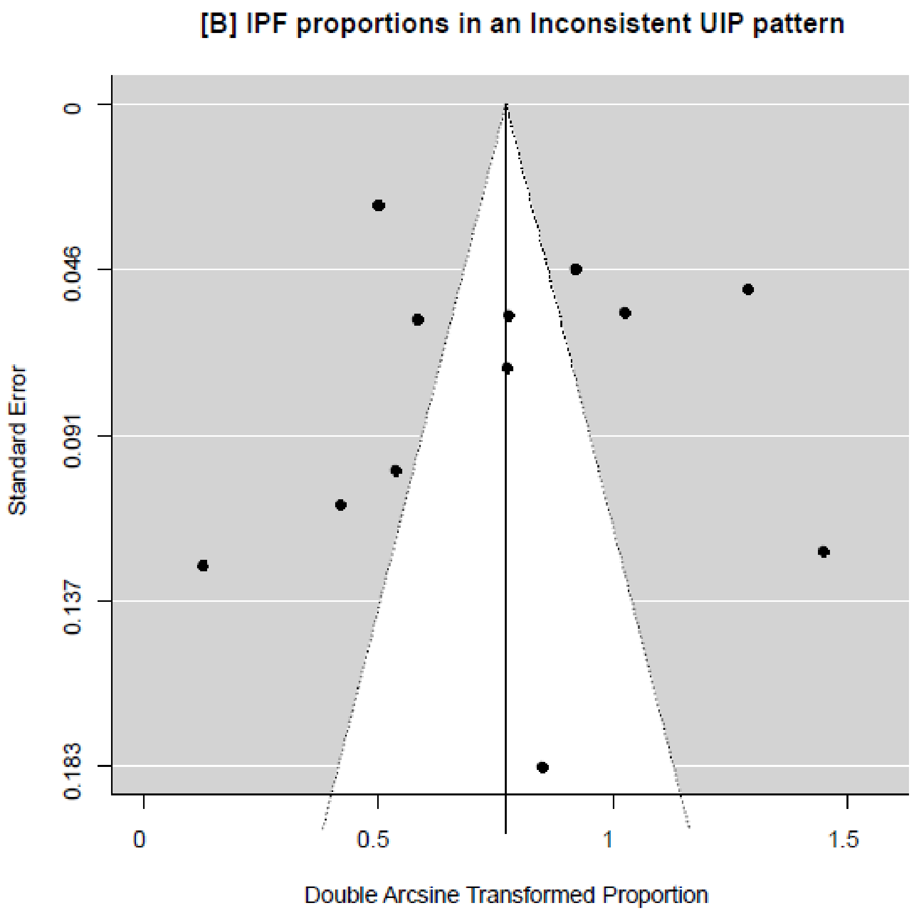

Supplementary data 5. Database search strategies

OVID/MEDLINE Search Strategy

1. Interstitial pneumonia. mp.

2. Usual interstitial pneumonia.mp.

3. Idiopathic pulmonary fibrosis.mo. or exp Idiopathic Pulmonary Fibrosis/

4. exp Tomography, X-Ray Computed/ or ct.mp.

5. pattern.mp. [mp=title, abstract, original title, name of substance word, subject heading word, keyword heading word, protocol supplementary concept word, rare disease supplementary concept word, unique identifier, synonyms]

6. 1 or 2 or 3

7. 4 or 5

8. 6 and 7

9. limit 8 yr=″2010-2017″

10. 8 and 9

EMBASE Search Strategy

#1. Usual AND interstitial AND pneumonia:ta,ab AND [embase]/lim AND [2010-2017]/py

#2. Interstitial AND pneumonia:ta,ab AND [embase]/lim AND [2010-2017]/py

#3. Idiopathic AND pulmonary AND fibrosis:ta,ab AND [embase]/lim AND [2010-2017]/py

#4. ct:ab AND [embase]/lim AND [2010-2017]/py

#5. Pattern

#6. #1 OR #2 OR #3 OR

#7. #4 OR #5

#8. #6 AND #7

Supplementary data 6. Summary of combination of HRCT pattern and result of histopathologic examination of surgical lung biopsy

		Histopathologic pattern				
		**UIP**	**Probable UIP**	**Possible UIP**	**Unclassifiable fibrosis**	**Not UIP**
**HRCT pattern**	**UIP**	IPF	IPF	IPF	IPF	Not IPF
	**Possible UIP**	IPF	IPF	Probable IPF	Probable IPF	Not IPF
	**Inconsistent with UIP**	Possible IPF	Not IPF	Not IPF	Not IPF	Not IPF

Definition of abbreviation: HRCT = high resolution computed tomography; IPF = idiopathic pulmonary fibrosis; UIP = usual interstitial pneumonia

* Multidisciplinary discussion is recommended in probable and possible IPF cases for the evaluation of potential sampling error and adequacy of technique of HRCT. Multidisciplinary discussion should involve experienced pulmonologists, radiologists, and pathologists who have experienced the diagnosis of interstitial lung disease.

Depending on whether the probable IPF was included in the IPF diagnosis or not, our analyses were performed based on the 2 following scenarios: scenario inclusive of probable IPF and scenario exclusive of probable IPF
